# The Impact of Beliefs on Health Information Social Sharing for Users: The Perspectives of Social Psychology and Information Technology

**DOI:** 10.3389/fpsyg.2022.891126

**Published:** 2022-05-04

**Authors:** Ruiqi Yao, Dongfang Sheng

**Affiliations:** School of Management, Shandong University, Jinan, China

**Keywords:** health information social sharing, UTAUT, normative beliefs, adoption intention, virtual health communities

## Abstract

With the integration and penetration of digitization into healthcare services, the comprehensive health industrial market is developing flourishingly. Users are fast-changing the way of health communication. This study investigates psychosocial and technological factors on health information sharing adoption through social sharing services. Based on the unified theory of acceptance and use of technology, social influence theory, and innovation diffusion theory, we developed a hypothesized model for health information social sharing adoption (HISSA), and dimensions of attitude beliefs, control beliefs, and normative beliefs were created. We conducted an empirical study on the adoption intention using a survey for data collection. The results were obtained from 375 valid questionnaires, and their interactions were tested and analyzed using PLS-structural equation modeling. Results implied that (1) social identity of normative beliefs was the most critical variable affecting behavioral intention, which revealed the importance of psychosocial factors; (2) behavioral intention was also determined by user's performance expectancy, facilitating conditions, subjective norm; (3) personal innovativeness had a negative effect on behavioral intention and positive effect on effort expectancy; and (4) effort expectancy and social identity had a positive effect on performance expectancy. This study advances the understanding of social sharing for health and provides references for the development of both virtual health communities and social sharing services to upgrade their products from user's behavior and psychology. This empirical research model may also be useful for researchers who are interested in user's health information behavior.

## Introduction

Entering the 21st century, the pursuit of human beings has changed from developing economy to caring for their health; health and medical informatization became constant popularity. With the advent of the mobile Internet era, social media was applied globally, especially in the healthcare context. Since the coronavirus disease 2019 (COVID-19) outbreak, social media played a vital role in disseminating information about the pandemic. Health information was posted in text and in more easily accessible and diffusible forms through social media (Li et al., 2018). Recently, an emerging service “social sharing” has greatly changed the nature and speed of health information interaction on social media. People were enabled to share health information to single or multiple communities through hyperlinks. For instance, a social media user could click the “share button” to share the popular science article “*The First Symptom of Novel Coronavirus Pneumonia May Be Olfactory or Taste Abnormalities*” from BBS.DXY.CN, a health information community, with her fellow wards on WeChat or QQ ZONE immediately. Since 2011, more than 50% of websites used social sharing tools around the world.

Health information social sharing provides opportunities for health knowledge among multiusers through multisensory communication (Moorhead et al., 2013). This benefits both the health community and its users. From the viewpoint of users, the share function of social sharing services maximizes health content and dissemination of information. Health information social sharing would strengthen the awareness of healthcare and the attention to their body condition. For example, people repost epidemic data to their circle to see if destinations are suitable for travel *via* health information social sharing and share action trajectory of suspected cases as a precaution (Pham et al., 2020). Health social sharing enables people without a professional background to distribute health information easily and rapidly, considering health information is characterized by professionalism and a high comprehension threshold. Thus, it has high use value and great potential to affect public health status. From the viewpoint of a health virtual community, health information social sharing diversified the way through which information content can be shared and converted its large base of inactive users into active ones, as well as let them keep surfing the web (Liu et al., 2018). Thus, health information social sharing promotes them to develop vigorously.

Previous literature has considered sharing health information from information technology (IT) perspective, mainly focusing on design and construction of the platform, influencing factors of health information sharing on social networking site (SNS) and virtual community (Fan et al., 2015; Zhang, 2018; Xia et al., 2021), etc. However, limitations still exist. Despite the extensive offer and the obvious potential benefits of health information social sharing, what motivates the adoption was still ambiguous. Health information social sharing was determined by the use of service and speed of adoption. Therefore, modeling user adoption behavior was necessary. Models about user adoption were established at the technological level in previous studies, but they were not comprehensive. We found that previous research models did not pay enough attention to the psychosocial factors on users and their sharing process, such as how individuals can better express and learn health knowledge through social sharing, how they are influenced by others when sharing health content, and why some users are willing to share with others and under what circumstances they are not willing to share. This results in the lack of sufficient explanatory power and one-sidedness of the research on health sharing adoption and its influencing factors that the limitations then caused frictions between theory and practice on the complex behavior psychology of users. Consequently, to fill the gap mentioned above, on the basis of existing studies, the theoretical investigation and model analysis of user adoption behavior in health information social sharing need to be further improved. This study aims to investigate factors on user's adoption intention of health information social sharing. The hypothetical model is proposed in dimensions of attitude beliefs, control beliefs, and normative beliefs to reveal the internal influence mechanism. This study advances the understanding of social sharing for users and reflects the combination of healthcare and emerging technologies, as well as provides insights for the development of both virtual health communities and social media.

This study is structured as follows. After the introduction, “Background and Research Hypotheses Development” section refers to the background and research hypotheses development. In the “Materials and Methods” section, we addressed materials and methods. The “Results” section is dedicated to the data analysis and results. This is followed by the “Discussion” section and the “Conclusion” section, respectively.

## Background and Research Hypotheses Development

### Background Literature

The Theory of Planned Behavior (TPB) is one of the most applied theories in the social and behavioral sciences. According to TPB, human action is guided by three kinds of considerations, namely, behavioral beliefs, normative beliefs, and control beliefs (Ajzen, 1991). Behavioral beliefs refer to beliefs about the likely outcomes of the behavior and the evaluations of these outcomes. Normative beliefs express the normative expectations of others, and control beliefs discuss the presence of factors that may facilitate or impede performance of the behavior and the perceived power of these factors. The TPB has received broad attention in the field of health sciences, environmental science, business and management, and educational research in recent years (Downs and Hausenblas, 2005; Yang, 2012; Adnan et al., 2017; Karimi and Makreet, 2020). Tomczyk et al. (2020) suggested that future research should apply more extensive measures of the TPB and other health behavior models, for example, regarding intentions or willingness.

**Figure 1:**
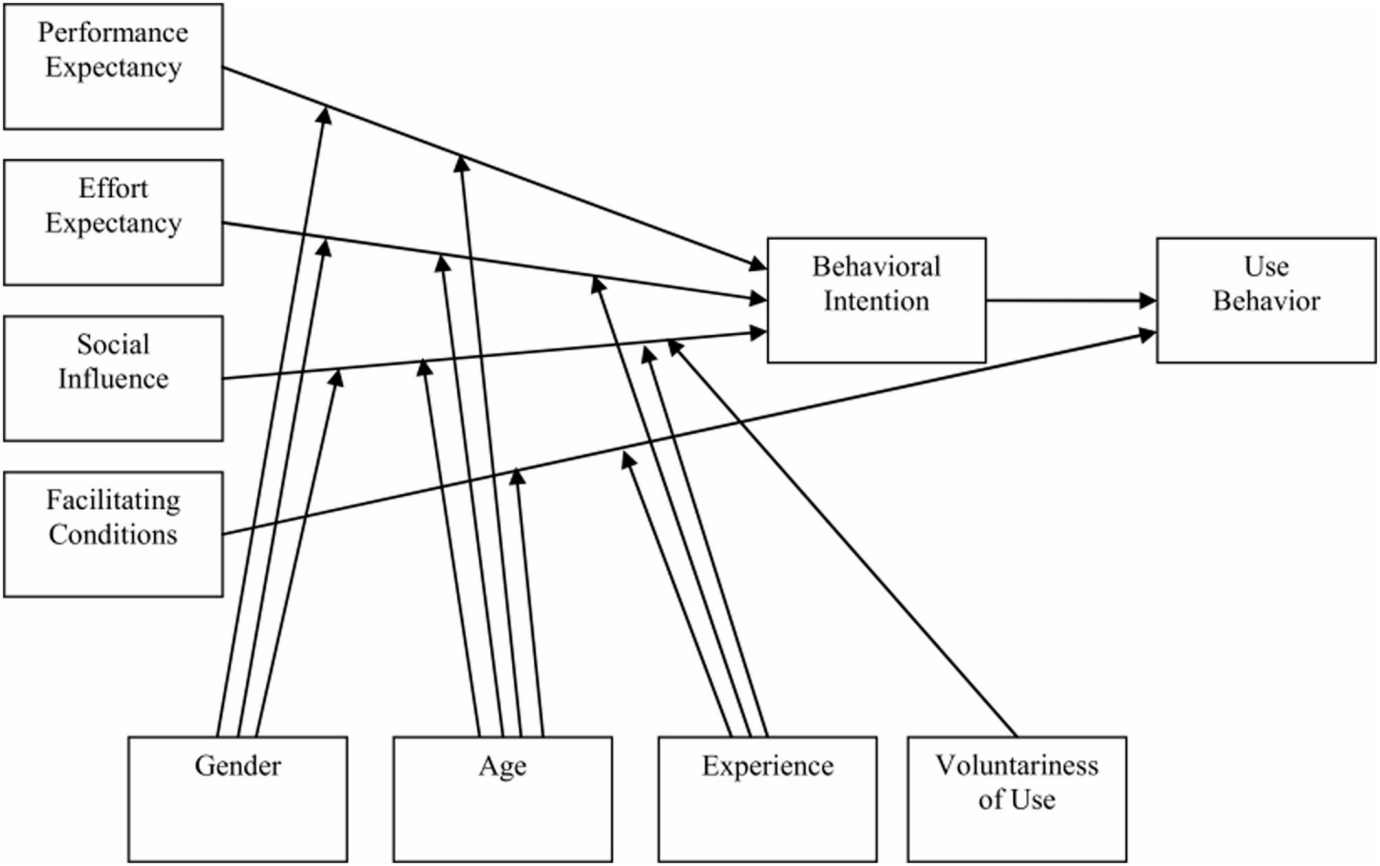
The unified theory of acceptance and use of technology.

Combining technology acceptance theories and behavior intention theories, Venkatesh et al. (2003) integrated 32 original constructs to get four component variables and four controlled variables. Then, a unified model named the Unified Theory of Acceptance and Use of Technology (UTAUT) was formulated (see Figure 1). As a popular theoretical model within the field of information technology, UTAUT includes constructs and relationships specific to an IT context, contributing to capturing the technology-related attributes and specific technology-generated environments (Castañeda et al., 2019). This model includes three variables (i.e., performance expectancy (PE), effort expectancy (EE), and social influence) that have positive effects on behavioral intention to use a technology, one variable [facilitating conditions (FCs)] that affects actual usage, and the adoption intention has a significant impact on user behavior (Venkatesh and Davis, 2000). Mpinganjira (2019) found that UTAUT can be appropriate for medical and health fields through research on factors of willingness to reciprocate in virtual health communities. Zhang et al. (2018) discussed factors on user medical information behavior in online health communities based on UTAUT. Although the model of UTAUT was originally devised to be used in the context of information technology acceptance as a universal model, which intended to be a timesaver for researchers to understand various theoretical models. After compiling 450 pieces of literature based on UTAUT, studies showed that the majority of research sought to combine UTAUT with other theories and constructs or used only partial constructs of this model (Williams et al., 2012). It seems that UTAUT cannot understand emerging scenarios any better. Venkatesh also pointed out that UTAUT was bound to have an even broader prospect for development. Thus, considering its characteristics, UTAUT should be modified in the context of health information and social sharing.

Social influence was a common social psychological phenomenon in people's lives, which refers to the use of the external role of individuals or groups to cause the change of individual thoughts, attitudes, and behaviors in a specific direction. Social Influence Theory (SIT) explained the antecedent variables of opinion formation and changes (Huang et al., 2013). The theory assumed that if attitudes based on different motives were adopted in different social impact situations, their qualitative characteristics and final behavior would be different (Kelman, 1958). Cheung and Lee (2010) used social networking sites together to explain in terms of the three social influence processes, namely, subjective norm (SN), group norm (GN), and social identity (SI). Even though, UTAUT integrated the construct of psychosocial factors from classic models and then develop social influence. Apparently, whether its definition or the questions, Venkatesh used to reflect that social influence remains in the category of SN and hardly contains its three mechanisms. Thus, the impact of social influence needs to be further improved.

Innovation diffusion theory (IDT) provides the theoretical basis for this study as well. It mainly describes the diffusion process of innovation in the social system. Rogers (2010) pointed out that individuals with high innovativeness had some behavioral characteristics such as actively searching for information, more public media exposure, and less dependent on the subjective evaluation of other members of the group. Many academics, practitioners have been focused on IDT after its proposition and spread-related research immediately, including research on the diffusion and process at the macro level and research on innovative adoption at the micro level (Wang and Chen, 2012). The innovativeness is an important factor to predict IT innovation adoption (Agarwal and Prasad, 1998). In recent years, IDT has been used to study users' adoption of new healthcare information technologies. Zhang et al. (2015) examined the major factors influencing patients' acceptance and use of the e-appointment service through the theoretical lens of Rogers' IDT. Jiang and Luan (2018) did a comparative study of Chinese and US pharmaceutical patents through the lenses of IDT approach and using network analysis and visualization techniques.

Combining the existing literature to sort out the detailed connotation and the application of the model and theory, according to the context of this study, influencing factors on health information social sharing adoption (HISSA) are divided into three dimensions, namely, attitude beliefs, control beliefs, and normative beliefs.

### Attitude Beliefs for Health Information Social Sharing

Attitude beliefs refer to a favorable or unfavorable attitude that behavioral beliefs (beliefs about the likely outcomes of the behavior and the evaluations of these outcomes) produce. We classified attitude beliefs into two variables due to their connotations and attributes. As one of the UTAUT's six main variables, performance expectation (PE) is a new concept integrating the perceived usefulness of Technology Acceptance Model (TAM) and the extrinsic motivation of the Theory of Motivation. Personal innovativeness (PI) is relevant to personal attitude; it refers to the degree to which a person believes that he/she is positively predisposed toward the use of new technologies (Agarwal and Prasad, 1998).

Performance expectancy is the perceived usefulness of adopting a system and the belief that the use of the adopted system will aid them in their job performance. (Brown et al., 2016) has been proved to significantly determine whether users accept the product in the context of social media. However, there is still less known about the impact of PE on social network technology and services, especially in sharing health information. Health information social sharing aims to simplify sharing procedures and improve health sharing efficiency. It would enhance their intention to use when thinking it can fulfill their demand of share, mainly reflected in how often they share valuable health information using social sharing services and how willing they are to communicate and share with their community members. Therefore, this study hypothesizes that:

Hypothesis (H1). PE positively affects the behavioral intention (BI) in health information social sharing.

The construct of PI was used to predict IT adoption intention, especially in the healthcare field such as patients' acceptance of consumer e-health, usage of mobile health applications, and school health education (Zhang et al., 2015; Gharaibeh et al., 2020). The adoption intention of innovativeness might be distinct due to the differences in innovativeness among individuals under the IDT (Brown et al., 2016). Karahanna and Chervany (2016) found out that people with a higher level of PI have a lower perception of difficulties in using the new system than other users. Another construct, the EE, reflects the new users' perception of system ease of use. Lewis and Sambamurthy (2003) suggested that PI had an influence on beliefs about ease of use in IT adoption. Walczuch et al. (2007) investigated the relationship between the personality attributes of employees and their use of IT support technology and found that PI had a significant influence on ease of use, employees with high PI felt less difficulty in using technology. Therefore, it can be supposed a relationship between the PI and EE in the health information sharing context. We expanded that relationship and considered that users with high PI have a much lower perception of use difficulty. In other words, their EE in social sharing services is high. Thus, we hypothesized that:

Hypotheses (H2a). PI positively affects BI in health information social sharing.
Hypotheses (H2b). PI positively affects EE in health information social sharing.

### Control Beliefs for Health Information Social Sharing

Based on TPB, control beliefs are relevant to perceived behavioral control and are predicted to provide the basis for perceptions of behavioral control. We grouped EE and FCs into control beliefs because they stress perceptions of behavioral control as well as individual control capabilities while using a new IT product. EE is one of the determinants of technology acceptance. FCs come from the variable of perceived behavioral control of TPB.

Effort expectancy refers to the level of ease in adopting the use of a technology system and recognized as a critical predictor of BI in the context of social networks (Wong et al., 2015). If the cost of learning and usage in health information social sharing exceeds a certain level, users would be likely to reduce or even give up their intention. On the contrary, if a new technology of health sharing service requires less effort to learn and understand the way of using it, the adoption intention would be higher (Chua et al., 2018).

According to UTAUT, perceived ease of use refers to the labor-saving degree of service, which has a significant positive impact on perceived usefulness. Chiu and Wang (2008) found that EE significantly affected users' intention to continue using the online learning system and had a profound impact on users' PE. Thus, it can be inferred that EE affects PE; users' PE would be increased, while they have a higher EE of the service. According to the context of this study, when the cost of learning and the cost of using health information social sharing service is low, users are likely to form the perception that “this service is helpful to me.” On the contrary, it may reduce user perception of the usefulness. Thus, we hypothesized that:

Hypotheses (H3a). EE positively affects BI in health information social sharing.
Hypotheses (H3b). EE positively affects PE in health information social sharing.

Facilitating conditions come from the variable of perceived behavioral control of TPB and is defined as the individual's belief about the support for the organization and technical facilities available when using the system. The study of Guan et al. (2012) has confirmed that FC could predict the intention of government officials to adopt microblog. Wilson and Lankton (2009) presented a rational-objective (R-O) model of e-health use that accounted for the effects of FC on predicting the use of e-health. According to this study, BI would ascend while people consider that they could get support for engaging in health information social sharing (e.g., devices and networks). On the contrary, their adoption intention would be decreased due to the lack of relevant conditions despite the demand for sharing health knowledge. Thus, we hypothesized that:

Hypothesis (H4). FC in health information social sharing positively affects BI.

### Normative Beliefs for Health Information Social Sharing

Ajzen pointed out in TPB that normative beliefs were beliefs about the normative expectations of others and motivation to comply with these expectations (Ajzen, 1991). According to the existing literature research results, we grouped media influence (MI), SN, and SI into normative beliefs.

Subjective norm refers to situations in which an individual's behavior is affected by the environment (Huang et al., 2008). Social psychologists pointed out that social networkers always tend to be consistent with the important someone (Pelling and White, 2009). SN significantly predicted intentions to engage in SNW use with intention significantly predicting behaviors. According to Zhao (2016), SN influences the continuance behavioral intention of Knowledge Question-and-Answer SNS users. According to the context of this study, users are more likely to be driven by others, and their sharing intentions related to healthcare are increased by then when their family and friends are sharing health information by using social sharing services or the important someone thinks they should do it. On the contrary, the idea of engagements with health information social sharing would be reduced or eliminated. Joseph and Jacob (2011) found in their study that SN had a strong impact on sharing knowledge intention. Thus, we hypothesized that:

Hypothesis (H5). SN positively affects BI in health information social sharing.

According to Rogers' research on IDT, adoption decisions are driven by some second-hand information sources (e.g., TV and journal). Laumer et al. (2010) considered the MI as objective norm (ON) to distinguish MI from SNs and listed them as two different types of normative beliefs, laying the foundation that MI can be the same rank as the SN and SI. The construct of MI may be preferably used in customers' adoption literature. A few studies on IT adoption have discussed the prediction abilities of media influence, as the study of Venkatesh and Brown (2001) pointed out that household PC adoption decisions would be influenced by the messages conveyed *via* the mass media. Similar studies also appear in the adoption of health information. Griffith et al. (2012) noted in their study that the mass media can affect the way that African American men obtain, process, and use health information as well. According to the context of this study, stakeholders (e.g., content providers, sharing service providers, and social media) would affect adoption decisions by conducting the service propaganda through various channels. Thus, we hypothesized that:

Hypothesis (H6). MI positively affects BI in health information social sharing.

Social identity derives from the user's interaction with others of his membership in a social group in the social networking environment. The motivation to establish and maintain SI would promote some online behaviors (Shen et al., 2011). Specifically, it has been suggested its importance of predicting the online social activity intention of SNS users besides SN in Cheung's study (Cheung and Lee, 2010).

Clément et al. (2001) have pointed out that users who collaborate with others to maintain their SI need communication support. Kwon and Wen (2010) have analyzed the relationship between SI and perceived ease of use in their research on social media adoption, and the empirical study indicates that SI has a significant positive impact on users' perceived ease of use. In the field of healthcare, psychologists suggest that SI support is exerted on attitudes, intentions, and health behavior (Chatzisarantis et al., 2009). According to the context of this study, it can be inferred that SI would influence the perceived usefulness while engaging in health information social sharing, i.e., SI is related to PE. Thus, we hypothesized that:

Hypotheses (H7a). SI positively affects BI in health information social sharing.
Hypotheses (H7b). SI positively affects PE in health information social sharing.

**Figure 2:**
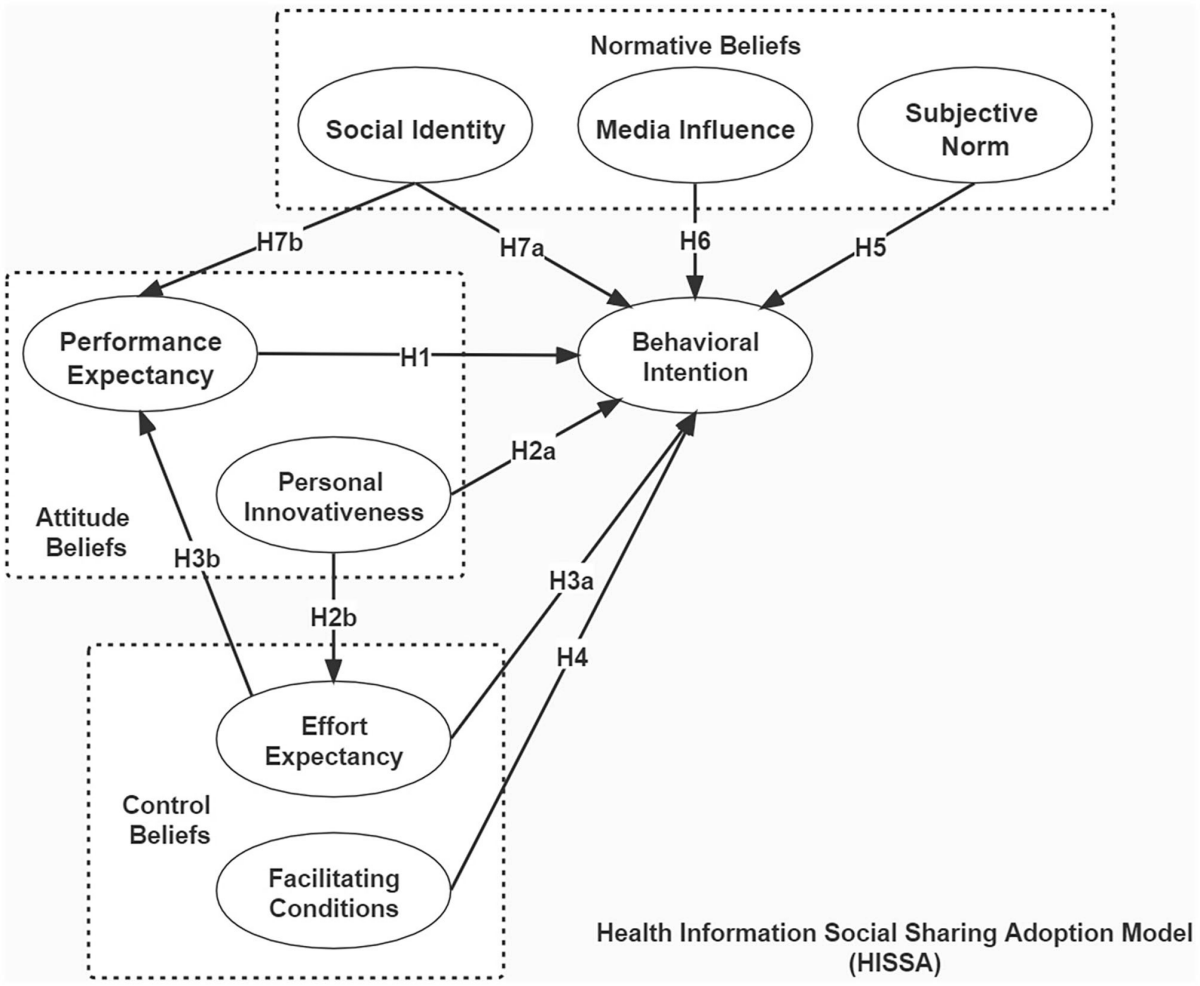
The hypothesized model of health information social sharing adoption (HISSA).

Based on the selection of the model variables above, this study inherits the core constructs of UTAUT and TPB considering the technological attributes of health information social sharing service and expanded it by combining the constructs and connotations of SIT and IDT considering that social sharing service was an emergent technological innovation with specific social attributes. Then, we developed a hypothesized model of health information social sharing adoption (HISSA) by integrating the interaction among dimensions of attitude beliefs, control beliefs, and normative beliefs and their constructs' effects on behavioral intention. The HISSA model is shown in Figure 2.

## Materials and Methods

### Data Collection

This study uses questionnaires, which were conducted both through network questionnaires and paper questionnaires. Convenience sampling and Snowball sampling were used to select participants. Online questionnaires were posted and collected on “Wenjuanxing” platform (http://www.sojump.com/). Paper questionnaires were sent out in public places with high people flow (e.g., hospitals and banks) and in colleges and universities. Small gifts were given to participants before filling out the questionnaires for their participation. To ensure the quality and authenticity of the returned questionnaire, IP address recognition was set to reject the repeated submission of online questionnaires from the same address. The time control method was used to ensure the attitude of the respondents serious as well. Additionally, invalid paper questionnaires such as including too many “uncertain” options or the same answer for all questions were rejected.

The questionnaire lasted 42 days. In total, 263 online questionnaires were returned, of which 212 were valid, and 177 paper questionnaires were returned, of which, 163 were valid after excluding the invalid questionnaires. Finally, 375 valid questionnaires were collected in total with an effective rate of 85.2%.

The categorical variables of the 375 valid questionnaires included gender, age, education, occupation, and their use of social media and social sharing. Each variable corresponded to “The 47th Statistical Report on Internet Development in China” published by CNNIC. Notably, 82.6% of participants had social sharing 5 times or more per month. Participant description is provided in Table 1.

**Table 1:** Participant characteristics.

**Variable**		***N***	**%**
Gender	Male	195	52.00%
	Female	180	48.00%
	Total	375	100%
Age	19 and under	75	20.00%
	20–29	101	26.93%
	30–39	111	29.60%
	40–49	53	14.13%
	Above 50	35	9.33%
	Total	375	100%
Education	Junior middle school or below	5	1.33%
	High school	98	26.13%
	College/University	205	54.61%
	Master or above	57	15.20%
	Total	375	100%
Occupation	Student	149	39.73%
	Government official	74	19.73%
	Enterprise staff	116	30.93%
	Freelance/unemployment/ others	36	9.60%
	Total	375	100%
How often have you used social media?	Hardly ever	23	6.13%
	Occasionally	50	13.33%
	Daily	242	64.53%
	Several times daily	60	16.00%
	Total	375	100%
How often have you used health information social sharing?	Never	5	1.33%
	Hardly ever	64	17.07%
	Occasionally	183	48.80%
	Daily	115	30.67%
	Several times daily	8	2.13%
	Total	375	100%

### Measurement Instrument

The measurement items of this study were mainly adapted from the previous studies where all have been applied with sufficient validity. We adapted them for PE, EE, FC, PI, SN, MI, BI, and SI according to the actual situation of the research object and context. The scale came from scientific design principles proposed by Churchill. The measurement items of each construct consisted of no less than two questions (Churchill, 1979). The formal questionnaire came in three major parts, namely, questionnaire instruction, basic information, and use survey of health information social sharing service. More specifically, the questionnaire instruction described questionnaire content, filing, and concept explanation. The basic information included personal information (i.e., sex, age, education, and occupation) and the online activity information that consisted of social media use frequency, health information social sharing frequency, source, content type, and channel. The use survey of health information social sharing service was made up of 37 measurement items. The 5-point Likert format from 1 to 5 (1 = totally disagree, 5 = totally agree) was adopted in this study.

Before the formal distribution of the questionnaire, the questionnaire was reviewed and modified by a group of experts including professors, associate professors, and Ph.D. students in the field of information systems, user information behavior, human-computer interaction, and social media to ensure that the measurement constructs, indicators, and questions were set up in a scientific way. Finally, the scale was formed with eight variables and thirty measurement items (see Table 2).

**Table 2:** Questionnaire survey items.

**Latent variable**	**Item**	**Related documents**
PE	PE 1: I think the social sharing tool allows me to share health information faster.	Venkatesh et al., 2003, 2012 Brown et al., 2016
	PE 2: I think the social sharing tool improves my health information sharing efficiency.	
	PE 3: I think the social sharing tool increases the possibility of finishing the sharing task.	
	PE 4: I think the social sharing tool is helpful to my sharing behavior.	
EE	EE 1: It's easy for me to learn how to use social sharing tools.	Venkatesh et al., 2003, 2012; Chua et al., 2018
	EE 2: I am clear about the use process of social sharing tools.	
	EE 3: It's easy for me to be familiar with social sharing tools.	
	EE 4: I think the social sharing tool is simple to handle.	
FC	FC 1: I have conditions to use social sharing tools (Wi-Fi, Mobile web, etc.).	Venkatesh et al., 2003, 2012; Wilson and Lankton, 2009
	FC 2: I have skills to use social sharing tools (cognition, practices, etc.).	
	FC 3: Social sharing tools are compatible with other software I use. (I can share information from online health community to my Wechat moments, micro-blogs, etc.)	
	FC 4: When I have trouble using social sharing tools, consulting others might solve it.	
PI	PI 1: When I hear about a new technology/software/service, I usually want to try it	Agarwal and Prasad, 1998; Sun and Jeyaraj, 2013 Gharaibeh et al., 2020
	PI 2: I'm always the one who uses new technology/software/services first, among my friends.	
	PI 3:I'd like to try new technology/software/services.	
SN	SN 1: People who are important to me think that I should use social sharing tools.	Bagozzi and Dholakia, 2002; Wu et al., 2011 Joseph and Jacob, 2011
	SN 2: People who are important to me would approve of my use of social sharing tools.	
	SN 3: People who influence me think that I should use social sharing tools.	
	SN 4: People whom I value his/her opinion think I should use social sharing tools.	
MI	MI 1:Some websites suggest people to use social sharing tools to share.	Venkatesh and Brown, 2001; Hong et al., 2008; Griffith et al., 2012
	MI 2:Some websites encourage people to use social sharing tools to share.	
	MI 3:I find that some websites are using social sharing tools.	
BI	BI 1: I will continue to use social sharing tools to share health information.	Venkatesh et al., 2003, 2012
	BI 2: I will always use social sharing tools to share health information.	
	BI 3: I will use social sharing tools frequently to share health information.	
SI	Imagine you are sharing health information to a group (moments, microblog, etc.) on some social sharing tool. Please evaluate:	Chatzisarantis et al., 2009; Cheung and Lee, 2010; Shen et al., 2011
	SI 1: The consistence between your self-identity and the image you project in the group.	
	SI 2: You are an important member of the group.	
	SI 3: You a valuable member of the group.	
	SI 4: Your level of intimacy with the community	
	SI 5: Your sense of belonging to the community	

The descriptive statistical analysis of all variables was shown in Table 3. The maximum and minimum of the variable reached 5 and 1, respectively, indicating that views of participants toward each item were mixed. The mean of most items can reach 3, which indicated the level of agreement of measurement items. More specifically, the variables EE, FC, MI, and BI have a higher mean, and considerable differences existed in PI and SN.

**Table 3:** Summary statistics.

**Variable**	**Indicator**	**Minimum**	**Maximum**	**Mean**	**Std**.	**Standard deviation coefficient**
PE	PE1	1	5	3.42	1.153	0.337
	PE2	1	5	3.37	1.144	0.339
	PE3	1	5	3.18	1.134	0.357
	PE4	1	5	3.49	1.067	0.306
PI	PI1	1	5	3.13	1.127	0.360
	PI2	1	5	2.93	1.140	0.389
	PI3	1	5	3.04	1.177	0.387
EE	EE1	1	5	3.56	1.083	0.304
	EE2	1	5	3.47	1.123	0.324
	EE3	1	5	3.61	1.052	0.291
	EE4	1	5	3.64	1.056	0.290
FC	FC1	1	5	3.75	1.098	0.293
	FC2	1	5	3.65	1.062	0.291
	FC3	1	5	3.66	1.044	0.285
	FC4	1	5	3.49	1.008	0.289
SN	SN1	1	5	3.18	1.065	0.335
	SN2	1	5	3.12	1.074	0.344
	SN3	1	5	3.10	1.069	0.345
	SN4	1	5	3.15	1.067	0.339
MI	MI1	1	5	3.49	1.079	0.309
	MI2	1	5	3.50	1.106	0.316
	MI3	1	5	3.64	1.065	0.293
SI	SI1	1	5	3.34	0.984	0.295
	SI2	1	5	3.05	1.043	0.342
	SI3	1	5	3.21	1.043	0.325
	SI4	1	5	3.20	0.982	0.307
	SI5	1	5	3.16	1.011	0.320
BI	BI1	1	5	3.57	0.997	0.279
	BI2	1	5	3.43	1.044	0.304
	BI3	1	5	3.42	1.059	0.310

### Data Analysis

This study uses Partial Least Squares Structural Equation Modeling (PLS-SEM) to simultaneously evaluate and analyze the structural model and the measurement model. The measurement model analysis and the structural model analysis were performed using SmartPLS 3.0.

## Results

### Measurement Model Analysis

#### Reliability Analysis

Cronbach's alpha and composite reliability (CR) were used for reliability analysis. Cronbach's alpha was used to assess the extent to which observed variables explain the latent variables they describe, and CR was used to examine the degree of internal consistency of the corresponding items (Shi et al., 2021). Preliminary runs demonstrated high reliability of the questionnaire when Cronbach's alpha and composite reliabilities of each latent variable were all above 0.7 (Hou, 2004). The results of reliability are given in Table 4. The values of Cronbach's alpha were all above 0.85, and the values of CR were all above 0.9, thus, confirming the good reliability for the model.

**Table 4:** Reliability analysis results.

**Variable**	**Cronbach's α**	**CR**
EE	0.93	0.95
FC	0.87	0.91
MI	0.87	0.92
PE	0.91	0.94
PI	0.87	0.92
SI	0.90	0.92
SN	0.94	0.96
BI	0.91	0.94

#### Validity Analysis

It was generally accepted that in a model with good convergent validity, loadings should be larger than 0.7 and the latent values of average variance extracted (AVE) should be all above 0.5 (Hair et al., 2006). Table 5 presents the analysis results on convergent validity, which indicates that the measurement model has good convergent validity.

**Table 5:** Convergent validity.

**Variable**	**Factor loadings**	**AVE**
EE	0.88–0.92	0.82
FC	0.76–0.89	0.72
MI	0.86–0.91	0.79
PE	0.86–0.92	0.79
PI	0.86–0.92	0.79
SI	0.75–0.88	0.71
SN	0.90–0.93	0.84
BI	0.91–0.92	0.84

The testing of discriminant validity was usually based on the standard proposed by Fornell and Larcker (1981). The square root of the AVE values of each latent variable are larger than the correlation coefficient (see Table 6). Furthermore, factor loadings and cross loadings were considered to test discriminant validity. Therefore, the questionnaire had good discriminant validity.

**Table 6:** Discriminant validity – the square root of average variance extracted (AVE)>latent variable correlation (LVC).

	**BI**	**EE**	**FC**	**MI**	**PE**	**PI**	**SI**	**SN**
BI	0.92							
EE	0.55	0.90						
FC	0.63	0.80	0.85					
MI	0.51	0.60	0.65	0.89				
PE	0.57	0.56	0.57	0.56	0.89			
PI	0.39	0.55	0.43	0.37	0.46	0.89		
SI	0.71	0.54	0.56	0.45	0.53	0.54	0.84	
SN	0.59	0.47	0.51	0.46	0.58	0.46	0.64	0.92

### Structural Model Analysis

We confirmed the fit of the structural model, and the path model is calculated by the partial least square method. As shown in Table 7, the coefficient of determination R-square of mediators: EE and PE were 0.297896 and 0.389365, respectively. The R-square of the dependent variable behavioral intention was 0.607335. They were closed or larger than the standard value of 0.3, which indicated good model interpretation. Meanwhile, based on the calculation of commonality and *R*^2^, the value of goodness-of-fit (GoF) was 0.595 and larger than the standard value of 0.36, thus, confirming the good overall adaptation degree of the model. Then, we used the bootstrapping approach to estimate the path coefficients.

**Table 7:** Communality and *R*^2^ of the path model after adding mediation variables.

**Variable**	**Communality**	***R* ^2^**
BI	0.84	0.61
EE	0.82	0.30
FC	0.72	
MI	0.79	
PE	0.79	0.39
PI	0.79	
SI	0.71	
SN	0.84	

**Figure 3:**
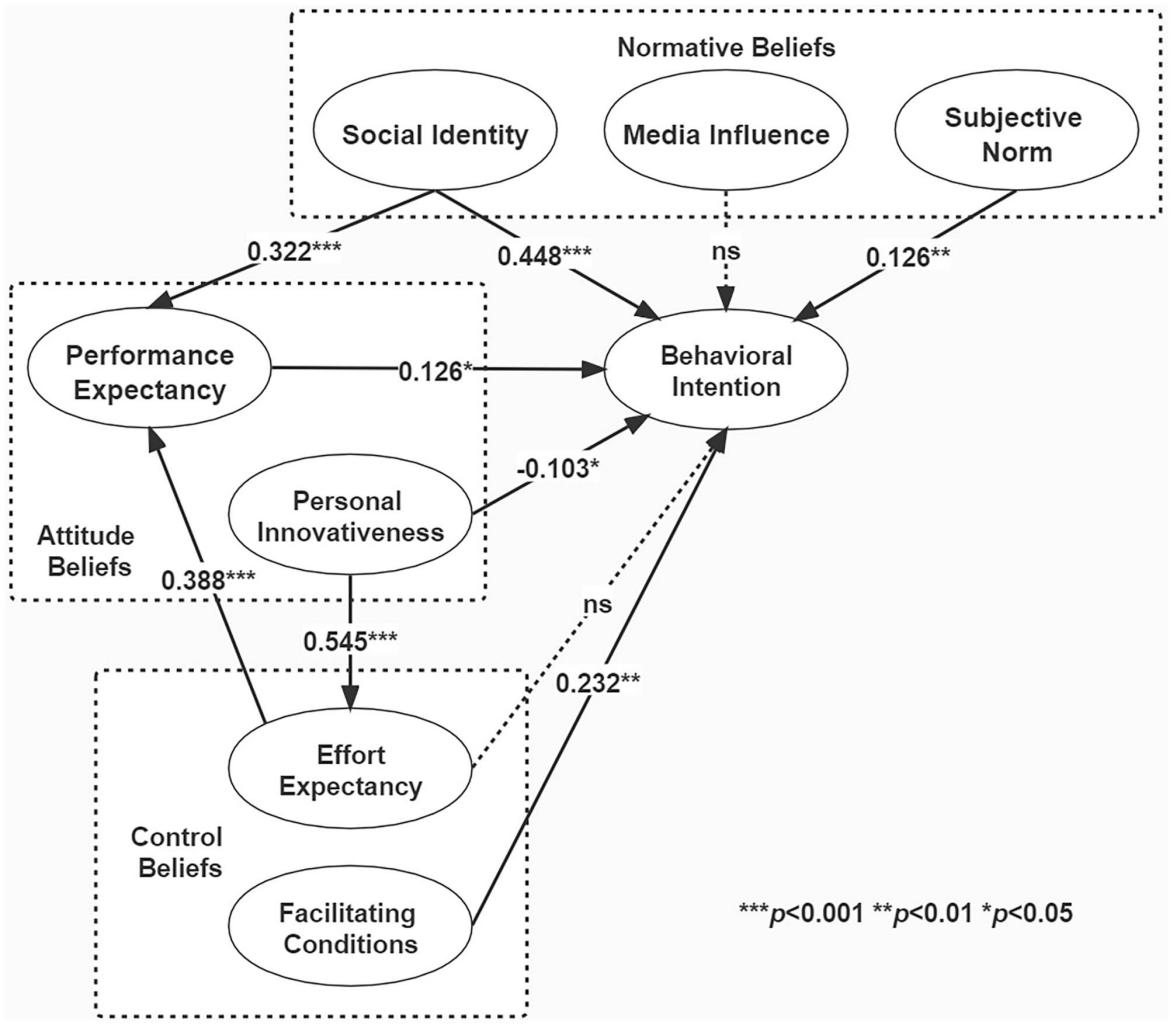
The results of structural model path analysis. ***indicates significance at the 0.001 level; **indicates significance at the 0.01 level, and *indicates significance at the 0.05 level.

The final model output is shown in Figure 3, and the hypothesis validation results are shown in Table 8. As shown in Figure 3 and Table 8, H2a, H3a, and H6 of the original hypothesis were not supported, while the rest of the hypotheses were supported.

**Table 8:** Path coefficients and hypothesis validation results.

**Hypothesis**	**Path**	**Path coefficients**	***T*-values**	**Significance**	**Hypothesis supported**
H1	PE→ BI	0.13	2.03	^*^	Yes
H2a	PI→ BI	−0.10	2.20	^*^	No
H2b	PI→ EE	0.55	12.21	^***^	Yes
H3a	EE→ BI	0.02	0.23	Insignificant	No
H3b	EE→ PE	0.39	6.38	^***^	Yes
H4	FC→ BI	0.23	2.94	^**^	Yes
H5	SN→ BI	0.13	1.74	^**^	Yes
H6	MI→ BI	0.06	1.02	Insignificant	No
H7a	SI→ BI	0.45	6.33	^***^	Yes
H7b	SI→ PE	0.32	5.63	^***^	Yes

*** *p < 0.001*;

** *p < 0.01*;

* *p < 0.5*.

## Discussion

### The Influence of Attitude Beliefs on the Adoption of Health Information Social Sharing

The results of the study showed that PE had a positive impact on BI, which supports H1. Most people agree that social sharing services could simplify health sharing processes and increase heath sharing efficiency. Lots of health information are long, professional, and have great instant dissemination value, such as information about coronavirus disease 2019 (COVID-19) epidemic prevention. Thus, it is important for users to be able to share them effectively and easily. Whether health information social sharing fulfill user's expectation would affect their behavioral intentions.

The path coefficient of PI on BI was −0.103, the lowest of all factors, which showed that H2a was not supported. The higher the PI, the lower the BI would be. This is contradictory to the previous research conclusions. One possible explanation is that most of the survey samples of this study are “non-rookie,” as well as experienced in using health information social sharing. The freshness of the service has faded for this type of users. Status Quo Bias Theory pointed out that people always prefer to keep their original state without making changes (Samuelson and Zeckhauser, 1988). Kim and Kankanhalli (2009) added more weight to the notion that the conservative users considered that the conversion cost was high when using sharing ways other than social sharing services. Thus, they were willing to conduct and adopt health information social sharing indeed.

Personal innovativeness had a significant positive impact on EE, which showed that H2b was supported. The more innovative a user is, the less he/she perceives the difficulty of health information social sharing, which corresponds to Roger's explanation toward users with different innovative characteristics.

The impact of attitude beliefs demonstrates that social sharing tools that can quickly share and disseminate real-time health information are more well-received. The design of social sharing tools should be easier to use on the basis of the user experience, correctly guide the user's operation, in the limited space and page information to easily find the share function, and simply and quickly achieve the aim (Xu and Tan, 2013). Moreover, humanized design is also essential. Zhang and Xiao (2017) note in their study that humanized social sharing services can improve mood and relieve stress. Moreover, the user's habits should be taken into account, and social sharing services should be carefully adjusted to avoid making changes that increase the difficulty of use.

### The Influence of Control Beliefs on the Adoption of Health Information Social Sharing

This study reveals that the effect of EE on BI was not significant. The path coefficient was 0.018499, which was lower than expected, so H3a was not supported. It is opposing to the result of Tan et al. (2012) who confirmed that EE has a strong impact on BI because apps that require significant efforts to use would discourage consumers from adopting it. Descriptive statistics of effort expectation variables in this study showed that the mean of effort expectation was higher than that of other variables, and the mean of each item was above 3.5. This result indicates that health information social sharing is generally user-friendly at present, as well as learning to operate and use it is simple for most users. Thus, the impact of EE on BI is no longer significant. In reality, people can skillfully share and repost the trajectory of suspected cases and close contacts to the WeChat and QQ groups, which helps the epidemic information diffusion. The effect of EE on PE was positive, so H3b was supported. Social sharing promotes health information exchange on social media, which makes people feel empowered and informed as well as enables them to take an active role in their daily health management (Zhang et al., 2021).

FC had a significant positive impact on BI, so H4 was supported. People are more likely to adopt social sharing services while they obtain more usage support. In reality, with the evolution of the Internet age, people could share health information on their smartphones anytime and anywhere. Lots of wise information technology of med (WITMED) apps and online health communities are with good compatibility (Yin et al., 2020), so people are allowed to share their contents by using social sharing tools.

The impact of control beliefs demonstrates that only health information with good quality, content, and usability can improve user's adoption and further promote the sharing behavior. Social sharing services should optimize the interface design on the basis of users' needs and lower the usage threshold, so that more users can exchange and share health information on the interface with comfortable design, complete functions, and convenient operation.

### The Influence of Normative Beliefs on the Adoption of Health Information Social Sharing

The result of this study shows that SN had a significant positive impact on BI, which showed that H5 was supported. The finding was consistent with Chong et al. (2012) and Taylor et al. (2011) of their past research studies, which suggested that SN plays a significant role in BI. The psychological principle in which SNs directly affect behavior intention was that as long as the actors thought the influencers expected their certain behavior, they would actively obey, even if they did not approve of this behavior or result. This can be concluded that the positive influence of family and friends can significantly increase the intention of health information social sharing. Kye et al. (2017) found that good subjective health was significantly associated with frequent information sharing, while family members were more likely to share health information. Especially, many older persons also have adult children living outside the home who could provide substantial support to the service if they could share useful healthcare knowledge to their parents and access specific, accurate information about their parents' health (Zulman et al., 2011).

This study reveals that the effect of MI on BI was not significant. The path coefficient was 0.056960, which was lower than expected, so H6 was not supported. In the descriptive statistics of variables, the mean of the three measurement items of MI was relatively high, which indicates that many users have obvious perceptions about the media direction of encouraging social sharing services. Xu et al. (2010) pointed out in their study that excessive promotion of mobile products in media has aroused users' mistrust, and the gap between usage experience and media promotion further strengthens users' prejudice against MI. One possible explanation is that people's suspicion of the media makes the adoption of health information social sharing not be positive publicity influenced.

Social identity had a significant positive impact on BI, so H7a was supported. Among all the constructs, SI has the highest ability to predict BI, indicating that the psychosocial factor was the most significant factor influencing the adoption of health information social sharing rather than the technological factor. Furthermore, SI had a significant positive impact on PE, so H7b was supported. This result shows that in essence, the intention of health information social sharing is to strengthen the connection with the “circle” and maintain the “image” of oneself in the circle more than to improve the efficiency of sharing. Therefore, factors that determine the adoption of SNA users are not only technical factors but more importantly whether the technology satisfies the social psychology behind the users. Zhang et al. (2017) believed that reputation, personal interests, and altruism would promote knowledge sharing in online health communities. For example, many online health communities design and implement an online reputation system as an incentive mechanism (Wang et al., 2020). The maintenance of reputation encourages people to pursue positive social identities in the hope of gaining other users' approval (e.g., likes, reposts, and comments) in order to enhance their self-esteem (Oh and Syn, 2015), a process that has the advantage of fulfilling social needs in an epidemic environment and effectively promoting the dissemination of health knowledge.

## Conclusion

This study investigated the impact of beliefs on user's adoption intention of health information social sharing in online communities. Based on the UTAUT, combined with SIT and IDT, the hypothesized model of HISSA was proposed, and the relevant influencing factors of attitude beliefs, control beliefs, and normative beliefs were analyzed by using PLS-SEM, finally obtaining the following meaningful findings. The results show that PE (H1), FCs (H4), SNs (H5), and SI (H7a) were significant predictors of behavioral intention to use health information social sharing services. By using a questionnaire method, we found out that psychosocial factors of normative beliefs were the most critical factor influencing user adoption intention, and most of the hypothesized relationships were manifested. The results implied that the model of HISSA can help understand the influencing factors on user adoption behavior in a health context.

### Theoretical and Practical Implications

The theoretical contribution of this study was to develop a new theoretical model in health information social sharing context by integrating both information technology and social psychology theory and investigates relationships between each independent variable and dependent variable (i.e., BI) in the model. This study then classified influencing factors on health information social sharing adoption into attitude beliefs, control beliefs, and normative beliefs. It enriches the theoretical structure of UTAUT and TPB and makes the research more persuasive and detailed. Additionally, the results examined good applicability and explanatory power of this model as well.

Based on the results, we argue that SI of normative beliefs is the most important predictor of user behavioral intention. The attention to psychosocial factors promotes this research process on healthcare information technology (such as mobile health apps and virtual health communities). In recent years, scholars have theorized that social psychology is critical in enabling the information sharing analytics process regarding healthcare IT because exploring the mechanism of health information social sharing is not independent from its context. For example, Lu et al. (2019) investigated what drives patients to share in online depression communities and found that a sense of shared identity, trust, and a sense of shared values had positive effects on their health information sharing behaviors. Jiang et al. (2020) argued that self-efficacy positively affected user sharing willingness in online health communities. Users' health information sharing was based on their interaction. The interaction effect between users was closely related to SI, communication, and understanding ability, judgment, and identification ability, which should be noticed to enhance self-efficacy. Despite these claims, evidence supporting the enabling importance degree of psychosocial factors has yet to be discussed in heath information sharing literature. Therefore, this article supports these claims and revealed that psychosocial factors are more significant than technological factors.

Furthermore, this study investigated the relationships between independent variables (i.e., PI-EE, EE-PE, and SI-PE) in the HISSA model that are scarcely mentioned in previous studies but important for a comprehensive understanding of the use of social sharing services. The results also have reference value for understanding the adoption, use, and sustainable use of information technology in the information sharing process and extend online knowledge sharing literature.

From the practical perspective, the results of this study are worthwhile not only for the researchers but also for those involved in the health information social sharing service and online health communities. Users are the principal part of online activities and play an important role in value co-creation through health information sharing. This study takes users as the research subject, helps understand their social sharing for health, and provides references for social sharing service providers. For example, since the SN and SI are two strong motivations for the user's behavioral intention, stakeholders of the social sharing service and virtual health communities can lean more on group or circle leaders to facilitate the dissemination of health information. Furthermore, owing to the development of big data and Internet technology over the years, the cost of health information seeking and sharing has been lowered significantly. Individuals have already realized the rapidness and convenience of health information social sharing services, so they are more likely to accept related apps and use them frequently. For this reason, this study helps people to improve their understanding of self-sharing behavior and raise the quality of health Information seeking, processing, and sharing. It provides the practical implication to improve people's health literacy and self-healthcare attention of the whole society.

### Limitations

In this study, the hypothesis that PI has a positive impact on BI is not supported. In the following study, we will consider increasing the sample size and introducing the time dimension to further investigate the impact of PI on the adoption intention of health information social sharing at different stages of innovation diffusion.

Studies have pointed out that the prediction of adoption intention of UTAUT was different or even contradictory under different cultural backgrounds. The following study will collect sample data in the context of Western culture in order to conduct a comparative study on factors of cross-cultural health information social sharing adoption.

## Data Availability Statement

The raw data supporting the conclusions of this article will be made available by the authors, without undue reservation.

## Ethics Statement

The studies involving human participants were reviewed and approved by School of Management, Shandong University. Written informed consent to participate in this study was provided by the participants' legal guardian/next of kin.

## Author Contributions

DS contributed to the conception and design of the study, reviewed, and edited the manuscript. RY analyzed and examined the data and wrote the original draft of the manuscript. All authors have read and agreed to the published version of the manuscript.

## Funding

This research was partially supported in the collection, analysis, and interpretation of data by the National Natural Science Foundation of China under Grant No. 71904106, the National Natural Science Foundation of China under Grant No. 72134004-1, the Ministry of Education of Humanities and Social Science project of China under Grant No. 19YJC870019, the China Postdoctoral Science Foundation under Grant No. 2018M632688, and the Social Science Foundation of Shandong Province under Grant No. 18CHLJ40.

## Conflict of Interest

The authors declare that the research was conducted in the absence of any commercial or financial relationships that could be construed as a potential conflict of interest.

## Publisher's Note

All claims expressed in this article are solely those of the authors and do not necessarily represent those of their affiliated organizations, or those of the publisher, the editors and the reviewers. Any product that may be evaluated in this article, or claim that may be made by its manufacturer, is not guaranteed or endorsed by the publisher.
